# Dynamic analysis of physiological indices and transcriptome profiling revealing the mechanisms of the allelopathic effects of phenolic acids on *Pinellia ternata*

**DOI:** 10.3389/fpls.2022.1039507

**Published:** 2022-10-18

**Authors:** Zhigui He, Yanfeng Wang, Yan Yan, Shaowei Qin, Huan He, Renjun Mao, Zongsuo Liang

**Affiliations:** ^1^ School of Leisure and Health, Guilin Tourism University, Guilin, China; ^2^ College of Life Sciences, Yan’an University, Yan’an, China; ^3^ College of Life Sciences, Northwest A&F University, Yangling, China; ^4^ Shaanxi Key Laboratory of Chinese Jujube, Yan’an, China; ^5^ College of Life Sciences and Medicine, Zhejiang Sci-Tech University, Hangzhou, China

**Keywords:** *Pinellia ternata*, allelopathic effect, phenolic acids, antioxidant enzyme activity, reactive oxygen species, transcriptome, continuous cropping obstacles

## Abstract

*Pinellia ternata* (Thunb.) is a famous traditional Chinese medicine with high medicinal value, but its culture is strongly hindered by the continuous cropping obstacles (CCO) which are tightly associated with allelopathic effects. Deciphering the response mechanisms of *P. ternata* to allelochemicals is critical for overcoming the CCO. Here, we elucidate the response of *P. ternata* to phenolic acids treatment *via* physiological indices, cellular approaches, and transcriptome analysis. Phenolic acids showed a significant effect on the growth of *P. ternata* seedlings, similar to the phenotype of continuous cropping. Cellular analysis demonstrated that phenolic acids remarkably induced root cell death. Physiological analysis revealed that phenolic acids induced the overaccumulated of H_2_O_2_ and $O_{2}^{-}$ in root cells. However, two exogenous antioxidants (L-ascorbic acid and β-gentiobiose) aid in the scavenging of over-accumulated H_2_O_2_ and $O_{2}^{-}$ by promoting the antioxidant enzyme activity such as superoxide dismutase (SOD), ascorbate peroxidase (APX), and catalase (CAT). Transcriptome analysis demonstrated that differentially expressed genes (DEGs) related to the cell wall degeneration and reactive oxygen species (ROS) metabolism were upregulated by phenolic acid treatment. In addition, downregulated DEGs involved in sucrose and starch metabolism and phenylpropanoid biosynthesis pathways decreased the key metabolites contents. Taken together, phenolic acids caused root cell death by inducing the overaccumulation of H_2_O_2_ and $O_{2}^{-}$ , and L-ascorbic acid and β-gentiobiose effectively alleviated ROS stress. The present study elucidates the underlying mechanism of the allelopathic effect of phenolic acids, offers valuable information for further understanding the mechanism of CCO, and could contribute to improving guidance for further *P. ternata* production.

## Introduction

Continuous cropping is a cultivation pattern in which a certain crop is consecutively planted in fields that have previously cultivated the same or similar species (Zhu, 2012; Li et al., 2019). This cultivation pattern could induce continuous cropping obstacles (CCO), also known as the consecutive monoculture problem or replant failure. CCO leads to serious reductions in crop yield and quality, which causes a serious of problems, such as frequent soil-borne diseases, loss of land fertility, and the deterioration of soil quality. The mechanisms of CCO are very complex and involve many factors, such as plant rhizosphere allelochemicals, microbial populations, and soil physical and chemical characteristics. These various factors are tightly interrelated, which can present challenges in experimental design.

CCO showed a significant effect not only on the cultivation of crops but also on medicinal plants. Moreover, rhizome medicinal materials suffer severely from CCO and most of them are staple medicinal materials with high economic value and large market demand. In China, more than 70% of Chinese medicinal materials using rhizomes face CCO, such as *Rehmannia glutinosa* (Zhang et al., 2019), *Radix pseudostellariae* (Wu et al., 2020a), and *Panax quinquefolius* (Li et al., 2021). With increased planting years, plant growth weakens, serious diseases and pests occur, and the quality and yield of medicinal materials are reduced, even to the point of leading to a lack of harvest. What is more worrisome is that CCO has restricted the sustainable development of traditional Chinese medicine agricultural production and the sustainable use of cultivated land resources.

*Pinellia ternata* (Thunb.) Beri is a perennial herb that belongs to the Araceae family and Pinellia genus. *P. ternata* (“Banxia” in Chinese) is used as medicine with its tuber. It has the effects of reducing stress, mitigating vomiting, removing phlegm, and relieving coughing. (Chinese Pharmacopoeia Commission, 2020). *P. ternata* is the top ten most used traditional Chinese medicine and it has a large demand (Zhang et al., 2016). The tuber of *P. ternata* is the main raw material in many Chinese patent-medicine preparations. It is a staple medicinal material mainly utilized in China’s modern traditional Chinese medicine industry chain. Unfortunately, *P. ternata* is seriously affected by CCO, which can cause more than 80% plants death. The same land needs to be rotated or rested for more than five years to continue planting. Therefore, the CCO of *P. ternata* urgently needs to be solved.

Allelochemicals can cause allelopathic effects, which result in a series of soil ecological problems. Allelochemicals penetrate the plant rhizosphere through rain and fog leaching, root secretion, microbial metabolism and secondary metabolites released by the plant (Zhou and Wu, 2012; Cheng and Cheng, 2015; Zhang et al., 2019). In recent decades, evidence has shown that allelochemicals released from plants into rhizosphere soil play important roles in CCO. Studies on *P. ternata* also demonstrated that the allelochemicals could destory the structure of the microbial community, which is the main reason for the CCO of *P. ternata* (He et al., 2018; He et al., 2019b; Zhao et al., 2021).

Phenolic acids are important secondary metabolites in plants and can be secreted into the rhizosphere. Previous studies on rice, broad bean, and ginseng have proven that phenolic acids such as cinnamic acid, salicylic acid, and p-cinnamic acid have significant allelopathic effects (Guo et al., 2020; Ho et al., 2020; Ni et al., 2021; Chen et al., 2022). In addition, studies have shown that the exogenous antioxidants can alleviate the effects of allelochemicals on plants. L-ascorbate acid and β-gentiobiose, as commonly-used exogenous antioxidants, play an important role in alleviating plant damage (Takahashi et al., 2014; Yang et al., 2018; Shah et al., 2022). Studying the effect of exogenous antioxidants on the physiological indices of plants under stress is helpful to elucidate the allelopathic effect and the mechanism of CCO.

To date, hundreds of allelochemicals with allelopathic effects have been identified. Studies also indicated that the decline in rhizomes yield was related to the accumulation of phenolic acids in the rhizosphere (Bertin et al., 2003; Zhang et al., 2019; Xin et al., 2019). Allelochemicals can induce reactive oxygen species (ROS) accumulation (Yang et al., 2018), change the microbial community structure (Clocchiatti et al., 2021), and alter the photosynthesis efficiency (Jiang et al., 2019). At present, studies on the CCO of medicinal plants mainly use 16S rRNA and ITS sequencing methods (Wu et al., 2020a; Zhang et al., 2020; Tong et al., 2021) to identify rhizosphere microbial species and analyze the microbial community structure (Wu et al., 2017; Wu et al., 2020b; Tan et al., 2021). However, the importance of allelochemicals in medicinal plants has been overlooked. There are few studies on the mechanism by which allelochemicals affect the growth of medicinal plants. It is necessary to study the effects of allelochemicals on the growth of *P. ternata* on multiple scales to reveal the mechanism of CCO in *P. ternata*. However, to our knowledge, there is no information available on the phenolic acid effects on *P. ternata*.

In this study, we simulated the phenolic acid content in a continuous crop field and evaluated its effects on *P. ternata*. The objectives of this study were to (1) study the variation in plant morphology, cellular death, and physiological indices with phenolic acid treatment; (2) investigate the transcriptome profiles and identify candidate genes and pathways related to phenolic acid treatment; and (3) elucidate the underlying mechanisms of *P. ternata* CCO.

## Materials and methods

### Plant material and growth conditions

*P. ternata* tubers were collected from the standardization cultivation field of *P. ternata* in Qingshui, Gansu Province. The tuber surface was disinfected with 1% sodium hypochlorite for five minutes and planted in hole seedling tray. The planting soil was made with the ratio of nutrient soil:vermiculite:perlite = 5:3:2. The nutrient soil was purchased from PINGSTRUP (Denmark). The vermiculite and perlite were purchased from Red Grass Company (China). The tubers were cultured in a greenhouse under a 12/12 h photoperiod (day/night) with 2000 lx, 25°C/20°C temperature (day/night), and 50% relative humidity. *P. ternata* plants with the same growth trend and uniform size were selected for the experiment.

### The mixed phenolic acid (MPA) and exogenous antioxidant treatments

Eight phenolic acids (gallic acid, protocatechuic acid, vanillic acid, syringic acid, vanillin, syringaldehyde, ferulic acid, and chlorogenic acid), were purchased from Aladdin Chemistry Co., Ltd. (Shanghai, China, purity ≥98%). Phenolic acids were mixed and diluted to four different concentrations. The concentrations of MPA were based on the phenolic acids in the rhizosphere soil (10-20 cm depth) of *P. ternata* in the three-year replanted field. The detailed concentrations are listed in **Table 1**.

**Table 1:** The composition of different mixed phenolic acids used in the present study.

	Concentration A(M-Pa-A)	Concentration (M-Pa-B)	Concentration (M-Pa-C)	Concentration (M-Pa-D)
Gallic acid (mg·L^-1^)	18.92	9.46	4.73	0.95
Protocatechuic acid (mg·L^-1^)	15.74	7.87	3.94	0.79
Chlorogenic acid (mg·L^-1^)	44.50	22.25	11.13	2.23
Vanillin (mg·L^-1^)	39.76	19.88	9.94	1.98
Syringic acid (mg·L^-1^)	30.64	15.32	7.66	1.53
Vanillic acid (mg·L^-1^)	50.34	25.17	12.59	2.52
Syringaldehyde (mg·L^-1^)	1.34	0.67	0.34	0.07
Ferulic acid (mg·L^-1^)	47.98	23.99	11.99	2.40

L-ascorbic acid and β-gentiobiose were purchased from Solarbio BioTech (China, purity ≥98%), and dissolved in water and prepared into solutions with concentrations of 25.0 mg·L^−1^ and 50.0 mg·L^−1^, respectively. Two exogenous antioxidants were added to the M-Pa-B solution when they were used and were present as M-Pa+As and M-Pa+Ge.

The roots of *P. ternata* seedlings were washed with sterile water and cultured in a culture flask containing 100 mL of MPA at different concentrations. Hogland solution (1/2) was used as the control (CK). Each treatment had six repetitions. After 72 h of treatment, the wilting rate was measured. Wilted seedlings were identified as stems and leaves that drooped and could not be recovered after being placed into distilled water (Luo et al., 2019).


Wilting ratio(%)=(Wilted seedlings/all seedlings)×100;

Growth inhibition rate = (Fresh weight of seedlings before treatment - Fresh weight of seedlings after treatment)/Fresh weight of seedlings before treatment (Luo et al., 2019).

### Determination of root cell viability

We accurately weigh 100 mg of fluorescein diacetate (FDA) (Solarbio, Beijing, China) and dissolved it in 4 mL of acetone to prepare a 25 mg·mL^-1^ mother liquor, which was stored away from light at -20 °C. When in use, FDA storage solution was added to 0.65 mol·L^-1^ mannitol to obtain FDA dyeing solution in proportion. 10 mg of propidium iodide (PI) (Solarbio, China) was dissolved in 2 mL of 0.65 mol·L^-1^ mannitol to prepare a 5 mol·L^-1^ mother liquor. The FDA (15 μg·mL^-1^) and PI (5 μg·mL^-1^) mixtures were used to treat *P. ternata* seedlings.

*P. ternata* seedlings were hydroponically cultured and treated with M-Pa-B for 1, 2, 3, and 4 h. After that, the root tips of seedlings were washed three times with deionized water and then dyed with the FDA-PI in the dark for 10 min (Yang et al., 2018). The excess dye was washed away thoroughly with deionized water after dyeing. Ten root samples were collected from five individual plants at each time point. A laser confocal scanning microscope (Revolution, Andor, England) was used for observation and photography. The excitation and emission wavelengths are 488 nm and 630 nm, respectively.

### Determination of antioxidant enzyme activity

Fibrous roots were collected at 0, 12, 36, and 72 h for the determination of six antioxidant enzyme activities, i.e., superoxide dismutase (SOD), peroxidase (POD), ascorbate peroxidase (APX), catalase (CAT), dehydroascorbic acid reductase (DHAR), and monodehydroascorbate reductase (MDHAR). The antioxidant enzyme extract referred to our previously established method (Mao et al., 2019). Briefly, samples were ground in an ice bath in 20 mmol·L^-1^ phosphate buffer (pH = 7.4) to obtain the homogenate and then centrifuged at 4000 g for 12 min at 4°C. The antioxidant enzyme activity was measured by using an antioxidant enzyme kit (COMIM Biotechnology Company, China). All enzyme activities were measured in three biological replicates.

### Determination of H_2_O_2_, O_2_^·−^ and MDA contents

The supernatant which used to determine antioxidant enzyme activity was also used to determine the hydrogen peroxide (H_2_O_2_), superoxide anion ( $O_{2}^{-}$ ), and malondialdehyde (MDA) contents. H_2_O_2_ and MDA contents were measured by using the relevant kits (COMIM Biotechnology Company, Suzhou, China). The detailed processes were performed according to the manufacturer’s instructions.

### Library construction and sequencing

Fibrous roots treated with MPA were collected at 0, 6, 12, 24, 36, and 48 h and were used for transcriptome sequencing analysis. Root samples randomly collected from 10 plants were mixed as one sample. Samples were frozen in liquid nitrogen immediately after the surface was washed with distilled water. Library construction and sequencing were performed by Allwegene Technology Company (Beijing, China).

### *De novo* transcriptome assembly and functional annotation

*De novo* transcriptome assembly of the reads was performed using the Trinity method with the default parameters. The Gene Ontology (GO) (http://www.geneontology.org) and Kyoto Encyclopedia of Genes and Genomes (KEGG) (http://www.genome.jp/kegg/) database were used to obtain GO terms and KEGG pathways.

### Gene expression level and identification of DEGs

Differential expression analysis was performed with the DESeq2 R package to identify DEGs among sample groups. The criteria to identify DEGs were *P<* 0.05, false discovery rate (FDR)< 0.001 and |log_2_Ratio| > 1. GO enrichment analysis of DEGs was performed using the GOseq R packages (Götz et al., 2008). KOBAS (http://kobas.cbi.pku.edu.cn/) was used to test the statistical enrichment of DEGs in the KEGG pathways (Xie et al., 2011). Functional annotation of DEGs was performed based on GO functional enrichment. Pathway enrichment analysis was carried out using the KEGG database. Items with a *P*-values< 0.05 were considered to be significantly enriched according to two-tailed Fisher’s tests.

### Quantitative real-time PCR analysis (qRT−PCR)

The RNA as described for transcriptome sequencing was also used for qRT*−*PCR analysis. Twelve selected genes were used to perform qRT*−*PCR analysis. The detailed method was described in our previous study with some modification (Mao et al., 2019). The qRT*−*PCR was conducted by using the CFX96 system (BIO-RAD, USA). The PCR conditions were 95°C for 5 min, followed by 45 cycles of 95°C for 10 s, 60°C for 30 s, and 70°C for 30 s. *GAPDH* was selected as the reference gene (Wang et al., 2020). The 2^−ΔΔCt^ method was used to calculate the relative expression levels. Samples used for qRT*−*PCR analysis were carried out in triplicate. The primers are listed in **Supplementary Table 1**.

### Data analysis

Statistical differences between the control and treated groups with regard to plant growth, antioxidant enzyme activity, H_2_O_2_, $O_{2}^{-}$ , MDA contents, and qRT−PCR analysis were tested with analysis of variance (ANOVA) using the SPSS 22.0 (SPSS SOFTWARE, USA). A regression analysis between RNA-seq and qRT-PCR results was also conducted by SPSS software. Significantly different analysis was performed by using least significant difference (LSD) method. All data are presented as the mean ± standard deviation (S.D.). Figures were generated by GraphPad Prism 9.3.1 (GraphPad Software, U.S).

## Results

### MPA affects *P. ternata* seedling growth

**Figure 1:**
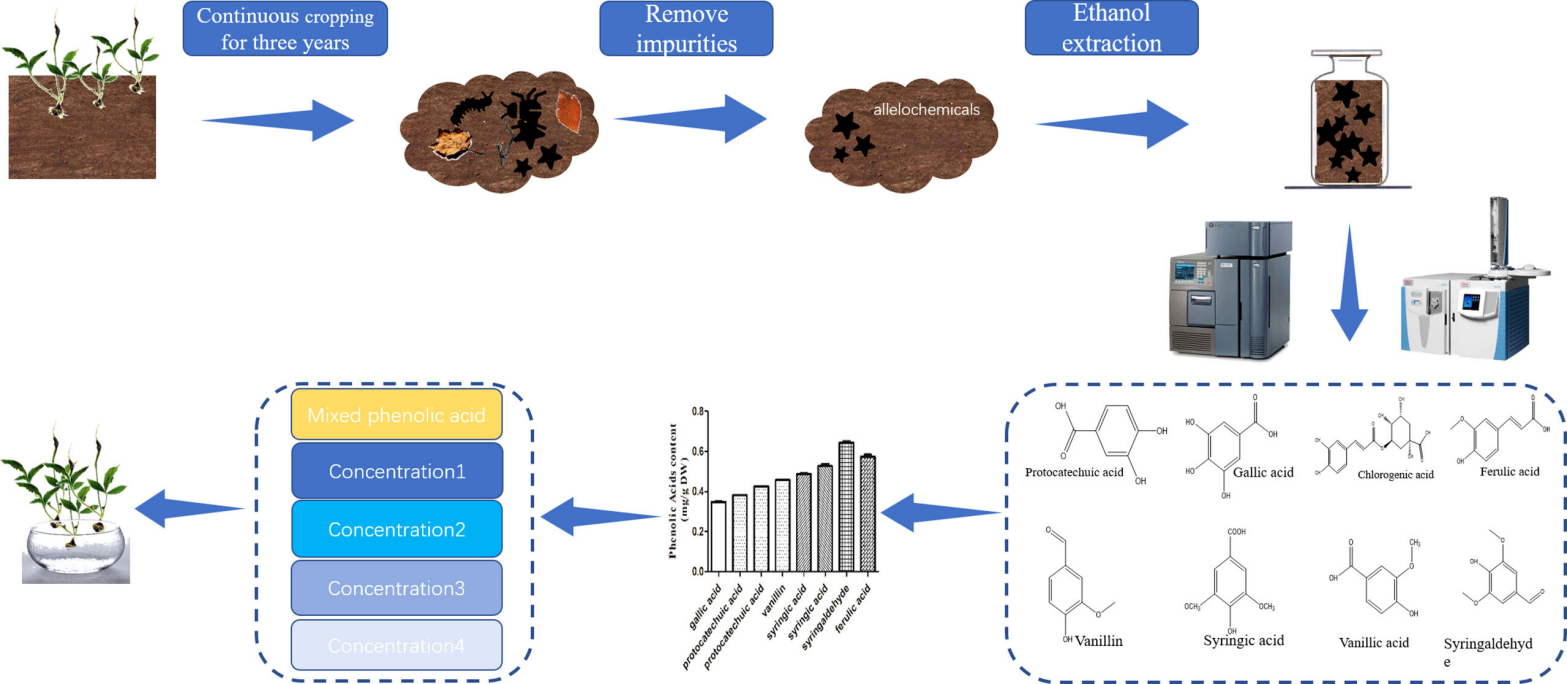
Extraction, separation and identification of the phenolic acids. The soil samples were collected from a three-year replanted field of *Pinellia ternata*. After removing the impurities, the chemical organic matter in the soil was extracted by ethanol and identified by LC−MS and HPLC. According to the contents of different phenolic acids identified in continuous cropping soil, the mixed phenolic acid solutions of different concentrations were prepared to treat *P. ternata* seedlings.

**Figure 2:**
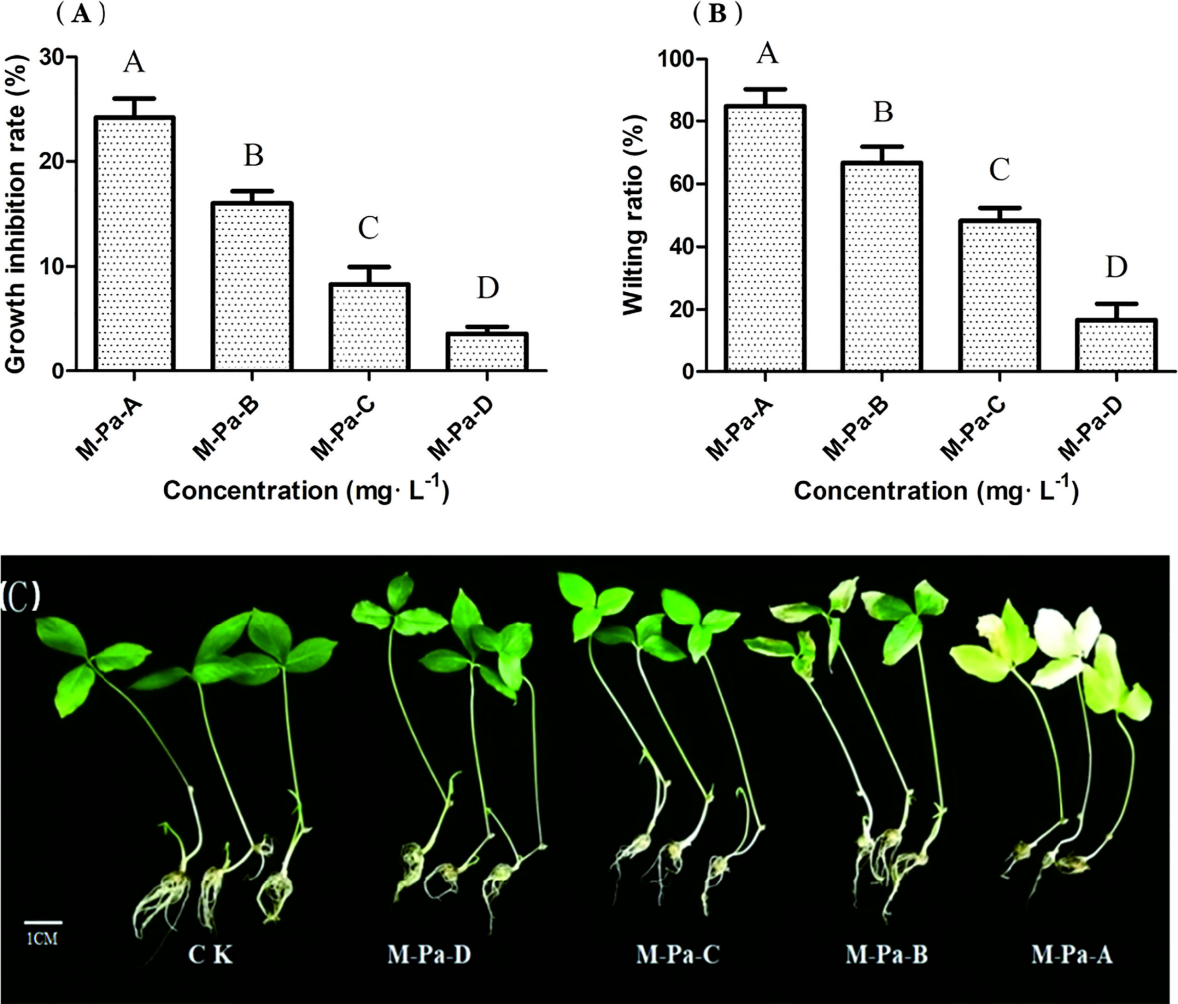
The effect of the mixed phenolic acid on *Pinellia ternata* seedlings. The growth inhibition rate **(A)** and wilting ratio **(B)** with the mixed phenolic acid treatment. The values are the mean ± SD (n = 6). The column diagrams of different letters are significantly different (least significant difference, LSD; P< 0.05; n = 6). The phenotype of *P. ternata* under four different concentrations of the mixed phenolic acid treatment **(C)**. Scale = 1 cm.

To study the types and contents of allelochemicals in the rhizosphere of *P. ternata* continuous cropping soil, we collected soil samples from the three-year replanted field of *P. ternata* and identified eight phenolic acids from the soil samples by liquid chromatograph−mass spectrometer (LC−MS) and high performance liquid chromatography (HPLC) (**Figure 1**). According to the phenolic acid concentration in the soil of *P. ternata* planted for three years, four concentrations of MPA were used to study the effects of phenolic acids on the growth of *P. ternata* seedlings. The results showed that MPA had a significant effect on *P. ternata* seedlings growth, which was related to the treatment concentration **Figure 2A**. The M-Pa-A treatment (the highest concentration) resulted in the maximum wilting rate and a significant growth inhibition (**Figure 2B**). The seedlings treated with M-Pa-A also exhibited leaf senescence. Subsequently, it was difficult for *P. ternata* seedlings to survive. M-Pa-B treatment led to the wilting of leaves, but the inhibitory effect was less obvious than that of M-Pa-A. The difference in leaf color between the M-Pa-D treatment and the control was not significant. The wilting rate and biomass decreased significantly with decreasing of MPA concentration (**Figure 2C**). *P. ternata* treated with phenolic acids showed a phenotype that was similar to that of CCO. Therefore, the concentration of M-Pa-B (M-Pa hereafter) was used for the subsequent experimental analysis.

### M-Pa induced fibrous root cell death

**Figure 3:**
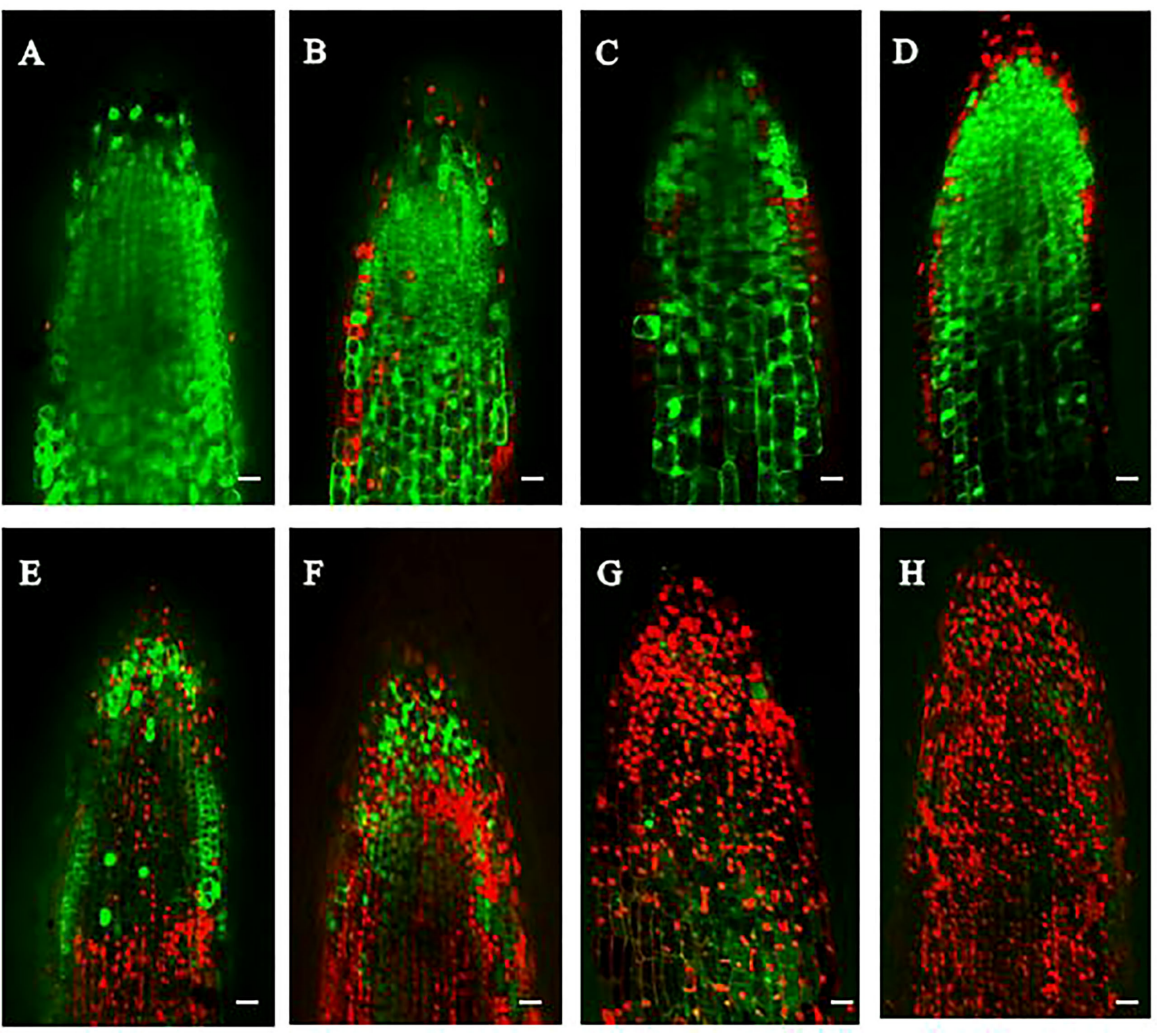
The mixed phenolic acid induced apoptosis of *Pinellia ternata* root tip cells. Fluorescein diacetate (FDA) and propidium iodide (PI) were used to stain living cells and cell membrane damaged cells with green and red, respectively. **(A–D)** show roots with the control at 1.0, 2.0, 3.0 and 4.0 h, respectively. **(E–H)** show root cells treated with the mixed phenolic acid treatment at 1.0, 2.0, 3.0 and 4.0 h, respectively. Scale = 100 μm.

To clarify the effect of M-Pa on *P. ternata* fibrous roots, the FDA-PI double fluorescence staining method was used. FDA stains living cells with green, and PI stains dead cells red. Confocal scanning microscope analysis indicated that more cells were stained by PI in the M-Pa treatment (**Figures 3A–D**) than that of control (**Figures 3E–H**). As shown in **Figure 3**, at the beginning of the treatment, the red-stained cells were mainly root epidermal cells (**Figure 3E**). As the treatment progressed, the number of red-stained cells continued to increase, and the proportion of red-stained cells increased accordingly. At 2 h, a small number of red-stained cells appeared in the root cap (**Figure 3F**). From 3 h, the cells in the elongation zone began to undergo apoptosis. After that, most of the cells were red-stained. After 4 h of treatment, most root cells treated with M-Pa were stained red by PI (**Figure 3H**). The number of red-stained cells increased significantly with the extension of treatment time. The results of microscope analysis demonstrated that M-Pa treatment induced root cell death.

### Effects of exogenous antioxidants on antioxidant enzyme activities

**Figure 4:**
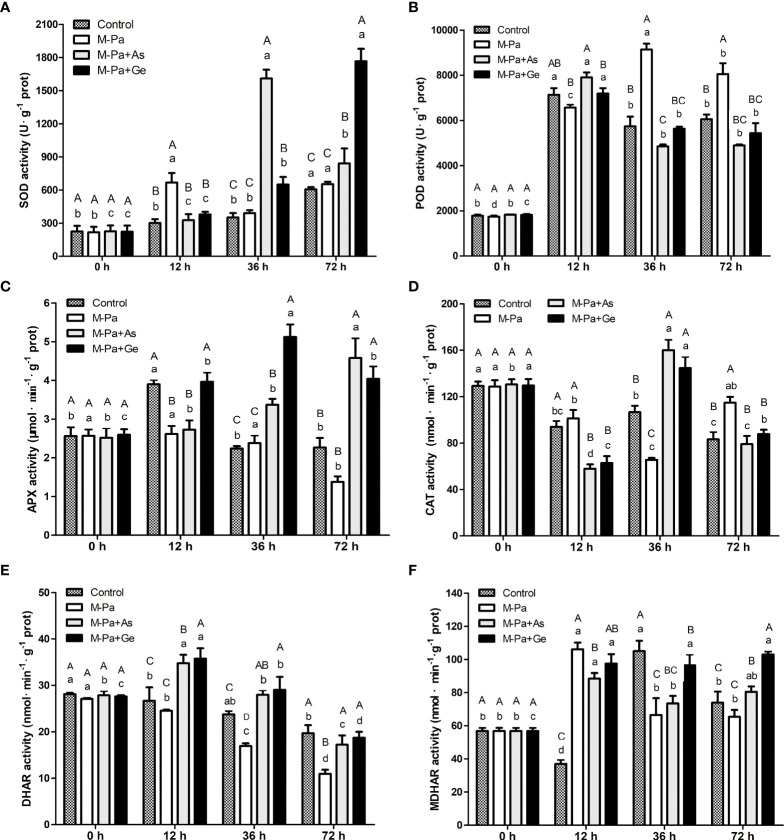
The variation patterns of antioxidant enzyme activity with the mixed phenolic acid (M-Pa) treatment. The activities of superoxide dismutase (SOD), catalase (CAT), peroxidase (POD), ascorbate peroxidase (APX), dehydroascorbic acid reductase (DHAR), and monodehydroascorbate reductase (MDHAR) are shown in **(A–F)**. Values are the mean ± SD (n = 3). Different capital letters indicate significant differences between the four treatments at the same time point. The lowercase letters indicate the differences in the same treatment among the four time points.

To analyze the effects of M-Pa treatment on *P. ternata* and evaluate the effect of L-ascorbate acid and β-gentiobiose, we measured the activities of six antioxidant enzymes at four time points. The results indicated that M-Pa induced oxidative stress, while two exogenous antioxidants reduced the oxidative damage. In the M-Pa treatment, SOD activity increased at 12 h and 72 h, showing a remarkable difference from the control (0 h) (**Figure 4A**). SOD activity in M-Pa+As treatment showed a significant jump at 36 h, reached the maximum value of 1609.94 U·g^-1^ and then decreased. It was retained at a high level at 72 h. In M-Pa+Ge treatment, the highest SOD activity was detected at 72 h. SOD activity of two exogenous antioxidant treatments increased significantly at 36 h and 72 h as compared to the control. At the same time point, exogenous antioxidant treatments showed a significant effect on SOD activity (**Figure 4A**). The SOD activity of M-Pa+As and M-Pa+Ge was significantly higher than that of the other treatments at 36 h and 72 h.

POD activity was remarkably increased at 12 h. In M-Pa treatment, POD activity increased significantly and reached the highest value at 36 h and remained at a high level at 72 h (**Figure 4B**). Notably, POD activity in M-Pa treatment showed significant differences among four time points. At 12 h, the POD activity of the M-Pa+As and M-Pa+Ge treatments reached the highest levels, which were 7190.00 U·g^-1^ and 7907.33 U·g^-1^, respectively (**Figure 4B**).

For APX activity, no significant variation was detected in M-Pa treatment except at 72 h, which showed a significant decreasing as compared to the control (**Figure 4C**). AXP activity in M-Pa+As treatment showed a continuous rising pattern. The highest value with 4.58 μmol^-1^·min^-1^·g was at 72 h. APX activity in M-Pa+Ge treatment increased first, reached the maximum value of 5.12 μmol^-1^·min^-1^·g at 36 h, and then decreased. At 36 h, APX activity in M-Pa+Ge was significantly higher than that in the other three groups (**Figure 4C**).

At 12 h, the CAT activity of M-Pa treatment was slightly higher than that of the control. Markedly lower CAT activity was detected in the exogenous treatments (**Figure 4D**). At 36 h, the CAT activity in the M-Pa+As and M-Pa+Ge treatments increased significantly and reached its maximum (160.01 and 144.64 μmol^-1^·min^-1^·g). M-Pa treatment significantly reduced CAT activity at 36 h. At 72 h, an elevated CAT activity was observed in the M-Pa treatment which was significantly higher than that in the two exogenous treatments (**Figure 4D**).

DHAR activity in M-Pa treatment exhibited a continuous decreasing pattern. However, the variation patterns of DHAR in M-Pa+As and M-Pa+Ge treatments were first increasing and then decreasing patterns (**Figure 4E**). The highest DHAR activity was observed at 12 h in the M-Pa+Ge treatment. DHAR activity in the M-Pa+Ge was slightly higher than that in M-Pa+As at the later three time points. Notably, DHAR activity in the M-Pa treatment was markedly lower than that in the M-Pa+As and M-Pa+Ge except at 0 h (**Figure 4E**).

MDHAR in the M-Pa treatment first significantly increased from 56.82 nmol^-1^·min^-1^·g at 0 h to 106.13 nmol^-1^·min^-1^·g at 12 h and then drastically decreased to 66.46 nmol^-1^·min^-1^·g at 36 h which was close to that of control (**Figure 4F**). The variation trend of MDHAR in the M-Pa-As treatment was similar to that of M-Pa. At 36 h and 72 h, the MDHAR activity in M-Pa+Ge was significantly higher than that of M-Pa (**Figure 4F**).

### Effect of exogenous antioxidants on H_2_O_2_ and O_2_^·−^ contents

**Figure 5:**
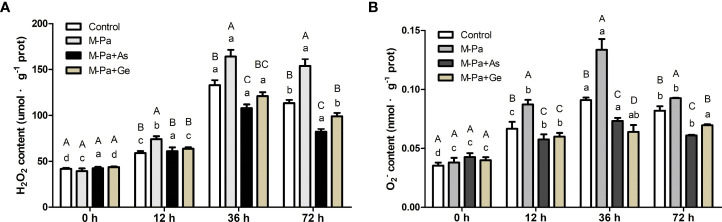
The variation patterns of H_2_O_2_ **(A)** and $O_{2}^{-}$ **(B)** contents with the mixed phenolic acid (M-Pa) treatment. Values are the mean ± SD (n = 3). Different capital letters indicate significant differences between four treatments at the same time point, while the lowercase letters indicate the differences in the same treatment among the four time points.

To elucidate the effect of M-Pa treatment on ROS over time in roots, their H_2_O_2_ and $O_{2}^{-}$ contents were measured. H_2_O_2_ content significantly accumulated with M-Pa treatment. Conversely, significantly lower H_2_O_2_ content was detected in the two antioxidant treatments than in the M-Pa treatment (**Figure 5A**). The H_2_O_2_ content was increased after 12 h of M-Pa treatment. At 36 h and 72 h, the H_2_O_2_ content was induced with M-Pa but significantly repressed in M-Pa+As and M-Pa+Ge treatments (**Figure 5A**). Strikingly, the H_2_O_2_ content in M-Pa+As and M-Pa+Ge decreased by 35.24% and 26.31%, respectively, compared with that in M-Pa. At 36 h, the H_2_O_2_ content of the two antioxidant treatments remarkably lower than that of M-Pa and the control. There was no significant difference between M-Pa-As and M-Pa+Ge, whereas a significant difference was observed at 72 h (**Figure 5A**).

The $O_{2}^{-}$ content increased at 12 h and 36 h with M-Pa treatment (**Figure 5B**). Except at 0 h, the $O_{2}^{-}$ content among the four treatments showed a similar pattern. M-Pa induced $O_{2}^{-}$ while exogenous antioxidants reduced the $O_{2}^{-}$ content. The highest level of $O_{2}^{-}$ content was detected at 36 h. Two exogenous antioxidant treatments were significantly lower than that of M-Pa (**Figure 5B**).

### Effect of exogenous antioxidants on MDA content

**Figure 6:**
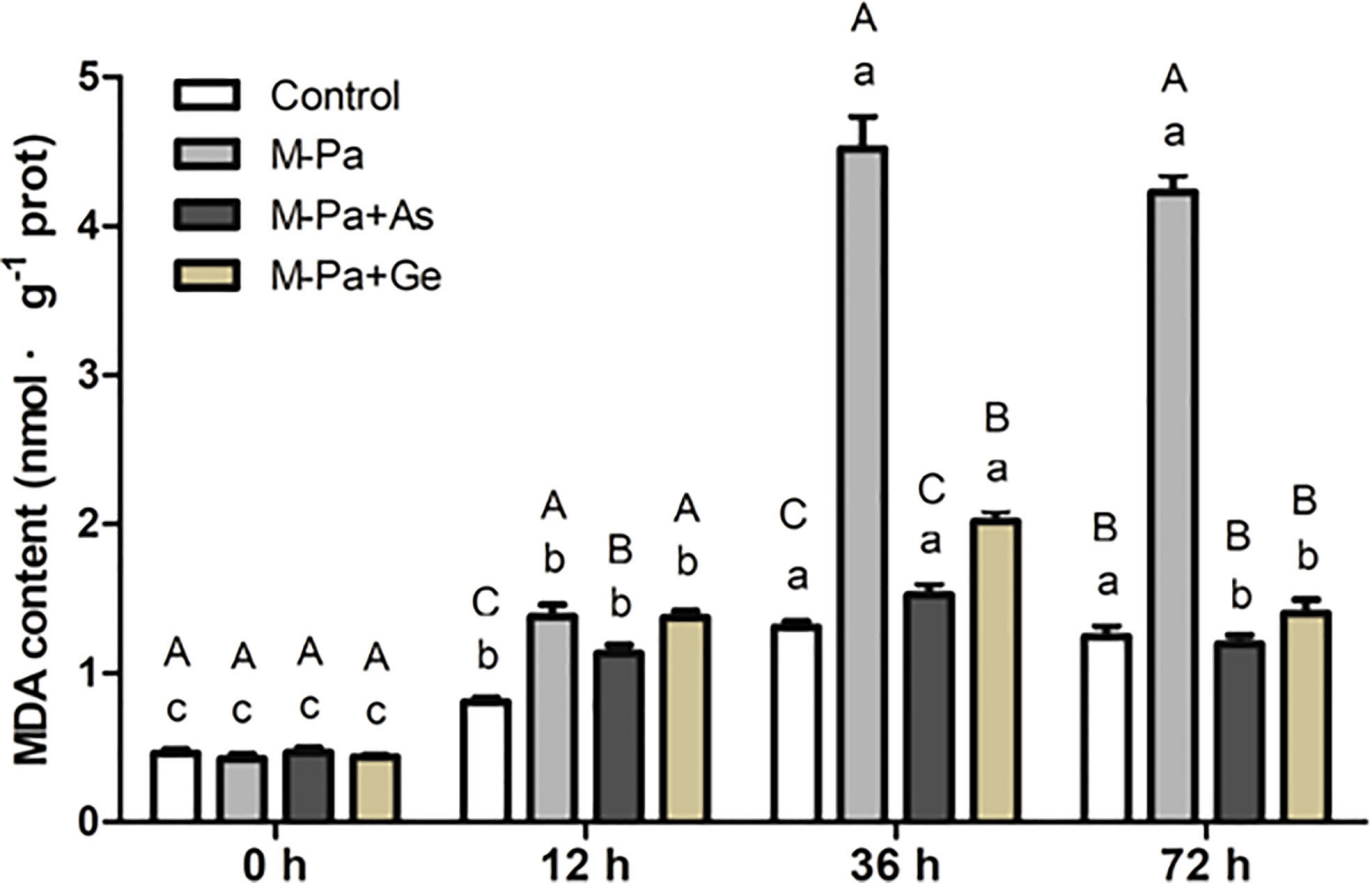
The variation patterns of MDA content with the mixed phenolic acid (M-Pa) treatment. Values are the mean ± SD (n = 3). Different capital letters indicate significant differences between the four treatments at the same time point, while the lowercase letters indicate the differences in the same treatment among the four time points.

To study the effect of M-Pa and antioxidants on cell membrane lipid peroxidation, MDA content was determined. The variation in MDA content first increased and then decreased. The maximum value appeared at 36 h. As shown in **Figure 6**, MDA content was increased significantly in M-Pa inducement at 36 h, reaching 4.52 nmol^-1^·g, which was remarkably higher than the other three treatments (**Figure 6**). The MDA content in the M-Pa+As and M-Pa+Ge treatments decreased by 66.7% and 55.3%, respectively, compared with that in the M-Pa treatment. At 72 h, the MDA content in the two exogenous antioxidant treatments was significantly lower than that in the M-Pa treatment (**Figure 6**).

### Transcriptome profiles

A total of 33 transcriptome databases were obtained. In total, 274.16 GB raw data and 262.86 GB of clean data were generated. The number of raw reads and clean reads were 914,930,998 and 876,187,106, respectively. More than 93.27% of the raw reads had a Q value≥30 (**Supplementary Table S2**). The GC content was 53.12% and the average error rate was 0.03%. The unigene annotation in the NR, NT, Ko, SwissProt, Pfam, and GO databases were 132,819, 59,433, 48,739, 75,613, 121,017, and 70,510, respectively (**Supplementary Table S3**). The unigenes sequence lengths of 500-1000 bp and 1000-2000 bp were the dominant patterns, accounting for 35.32% (91,035) and 22.01% (56,725) of all sequences, respectively. These parameters indicated that the transcriptome results were well reassembled and gained abundant data for further bioinformation analysis. Transcriptome data have been submitted to the SRA database (https://www.ncbi.nlm.nih.gov/sra/) under the accession numbers PRJNA791970. A total of 70,510 and 76,556 unigenes were annotated into 41 GO terms and 36 KOG classifications to identify their possible functions (**Supplementary Figure S1** and **S2**).

**Figure 7:**
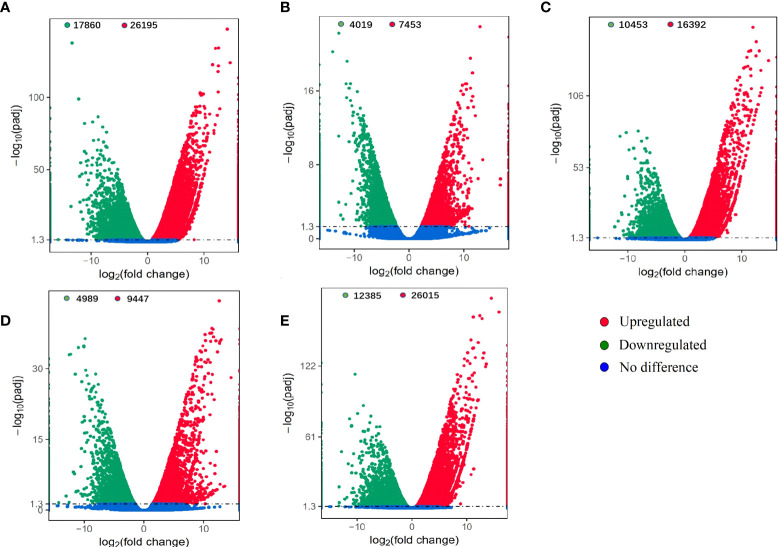
Differentially expressed genes (DEGs) identified between the mixed phenolic acid (M-Pa-B)-treated and CK groups. The comparisons of 6 h, 12 h, 24 h, 36 h and 48 h with CK are shown as TM6 vs. CK **(A)**, TM12 vs. CK **(B)**, TM24 vs. CK **(C)**, TM36 vs. CK **(D)**, and TM48 vs. CK **(E)**, respectively.

A total of 44,055, 11,472, 26,845, 14,436 and 38,400 DEGs were identified in the TM6 vs. CK, TM12 vs. CK, TM24 vs. CK, TM36 vs. CK, and TM48 vs. CK comparisons, respectively (**Figure 7**). The TM6 vs. CK comparison contained the largest number of DEGs (44,055), with 26,195 DEGs upregulated and 17,860 downregulated DEGs. The TM12 vs. CK comparison identified the minimum number of DEGs (11,472), among which 7453 were upregulated and 4019 were downregulated (**Figure 7**).

### Functional annotation and KEGG classification of the DEGs

**Figure 8:**
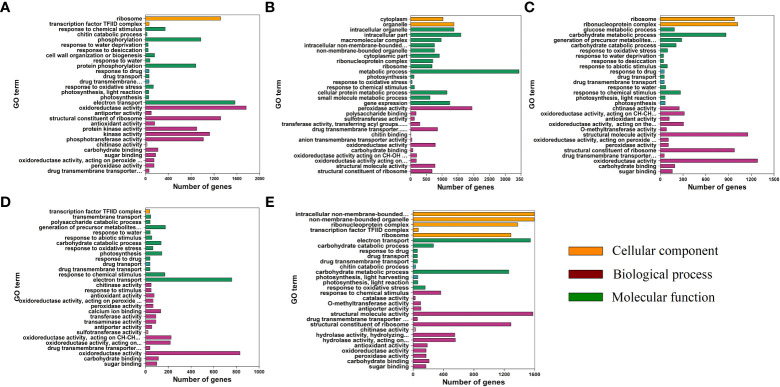
GO annotation analysis of DEGs in the TM6 vs. CK, **(A)** TM12 vs. CK, **(B)** TM24 vs. CK, **(C)** TM36 vs. CK, **(D)** and TM48 vs. CK **(E)** comparisons. The orange, green, and purple columns represent the cellular component (CC), biological process (BP), and molecular function (MF), respectively.

To reveal the function of the unigenes, GO annotation analysis was performed (**Figure 8**). For the biological process (BP) category, “metabolic process” was the most enriched in the TM12 vs. CK comparison. “Carbohydrate metabolic process” were the most enriched term in TM24 vs. CK and TM48 vs. CK comparisons. The other two comparisons were most enriched in “electron transport”. For the cellular component (CC) category, the most enriched terms in the TM6 vs. CK, TM12 vs. CK, and TM36 vs. CK comparisons were “ribosome”, “intracellular part”, and “transcription factor TFIID complex”, respectively. “Ribonucleoprotein complex” and “non-membrane-bounded organelle”was the most enriched term in the TM24 vs. CK and TM48 vs. CK comparisons. Noticeably, in all comparisons, DEGs were enriched in “response to oxidative stress” and “response to chemical stimulus” of BP. The “oxidoreductase activity “ in MF was enriched in all comparisons (**Figure 8**).

**Figure 9:**
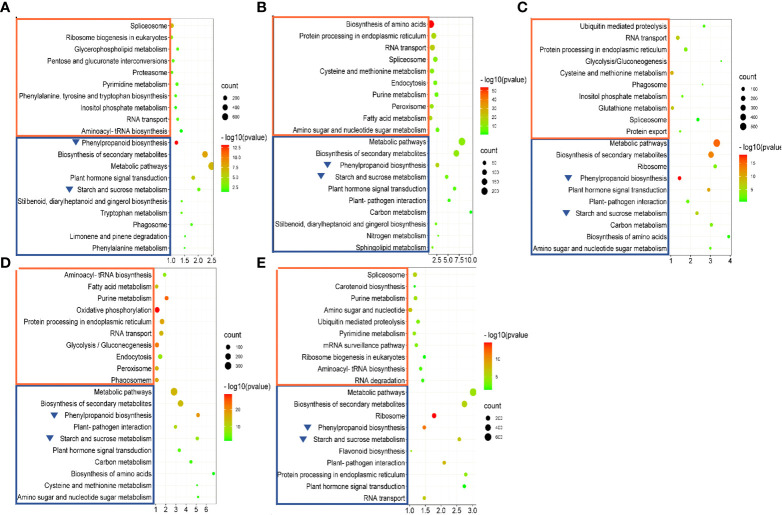
KEGG enrichment of differentially expressed genes (DEGs) in the comparisons of TM6 vs. CK **(A)**, TM12 vs. CK **(B)**, TM24 vs. CK **(C)**, TM36 vs. CK **(D)** and TM48 vs. CK **(E)**. The areas of bubbles indicate the number of enriched DEGs, while the color of bubbles indicates the Q value. The orange and blue rectangles indicate the KEGG pathways enriched with upregulated and downregulated DEGs. The blue triangle indicates the downregulated metabolic pathway identified in all five comparisons.

To determine whether these DEGs were involved in specific metabolic pathways, KEGG pathway analysis was performed. KEGG pathways enriched with upregulated DEGs included “spliceosome”, “RNA transport”, and “purine metabolism” (**Figure 9**). No specific pathways were enriched in any of the comparisons. “Spliceosome” was enriched in all comparisons except TM36 vs. CK. “RNA transport” was enriched in all comparisons except TM48 vs. CK. TM12 vs. CK, TM36 vs. CK, and TM48 vs. CK comparisons were all enriched with “purine metabolism”. Differentially, five pathways enriched with downregulated DEGs, “starch and sucrose metabolism”, “metabolic pathway”, “phenylpropanoid biosynthesis”, “plant hormone signal transduction”, and “biosynthesis of secondary metabolites”, were enriched in all comparisons (**Figure 9**). Combining the GO enrichment and KEGG pathway analyses, two significantly changed pathways, “starch and sucrose metabolism” (ath: 00500) and “phenylpropanoid biosynthesis” (ath: 00940), which may be important for elucidating the molecular mechanism of M-Pa treatment, were chosen for deeper study.

### DEGs involved in starch and sucrose metabolism

Starch and sucrose are the major products of photosynthesis and sustain cell metabolism and plant growth. The starch and sucrose metabolism pathway was enriched in all comparisons and some key enzyme genes were identified as DEGs. For example, TRINITY_DN14243_c0_g1_i1, encoding O-glycosyl hydrolase family 17 protein, was downregulated 4.37-, 3.80-, 2.57-, 2.36-, and 4.39-fold in the TM6 vs. CK, TM12 vs. CK, TM24 vs. CK, TM36 vs. CK, and TM48 vs. CK comparisons, respectively. TRINITY_DN11615_c0_g2_i1, which encodes trehalose-6-phosphate synthase 5 (TPS5), showed 4.13-, 3.68-, 3.47-, 2.87-, and 3.36-fold decreases in the TM6 vs. CK, TM12 vs. CK, TM24 vs. CK, TM36 vs. CK, and TM48 vs. CK comparisons, respectively. Similarly, glycosyl hydrolase family 32 proteins were also enriched with downregulated DEGs. TRINITY_DN11615_c0_g3_i3 showed 3.71- and 3.02-fold decreases in the TM12 vs. CK and TM24 vs. CK comparisons. Notably, TRINITY_DN97023_c0_g1_i1 encoding cell wall invertase 4 (cwINV4) was detected in all comparisons and exhibited 3.59-, 2.55-, 3.99-, 3.37-, and 4.47-fold decreases in the TM6 vs. CK, TM12 vs. CK, TM24 vs. CK, TM36 vs. CK, and TM48 vs. CK comparisons, respectively.

### DEGs involved in phenylpropanoid biosynthesis

Phenylpropanoid biosynthesis is another significantly enriched pathway in all comparisons. Some DEGs encoding key enzyme genes with downregulated patterns were detected. For example, caffeoyl-CoA 3-O-methyltransferase (CCoAMT) is one of the key enzymes in phenylpropanoid biosynthesis that catalyzes S-adenosyl-L-methionine to S-adenosylhomocysteine. TRINITY_DN8216_c0_g1_i12, which encoded *CCoAMT* gene, showed the downregulation with 3.31-, 4.42-, 2.71-, 3.70-, and 4.93-fold in TM6 vs. CK, TM12 vs. CK, TM24 vs. CK, TM36 vs. CK, and TM48 vs. CK comparisons, respectively. 4-coumarate: CoA ligase 1 (4CL1) is another key enzyme that catalyzes phenylalanine to monolignol. TRINITY_DN7979_c0_g1_i3, which was identified as a *4CL1* gene, was detected in all comparisons except TM12 vs. CK. It showed 1.93-, 2.63-, 3.07-, and 3.66-fold downregulation in TM6 vs. CK, TM24 vs. CK, TM36 vs. CK, and TM48 vs. CK comparisons. Furthermore, the expression level of cinnamate-4-hydroxylase (*C4H*) gene, encoded by TRINITY_DN105480_c0_g2_i3, TRINITY_DN105480_c0_g2_i2 and TRINITY_DN938_c1_g1_i6, in the TM24 vs. CK, showed 3.24-, 2.92-, and 1.90-fold downregulation. Additionally, the expression levels of TRINITY_DN8578_c0_g1_i4, encoding acyl-CoA synthetase 5 (ACOS5), were downregulated 6.46-, 5.68-, and 6.66-fold in the TM12 vs. CK, TM24 vs. CK, and TM36 vs. CK comparisons, respectively.

### ROS scavenging-related DEGs

Antioxidant enzyme genes are important parts of the antioxidant system and are highly associated with ROS scavenging. We determined the expression patterns of antioxidant enzyme genes and ROS-related genes. In line with the physiological data, antioxidant enzyme genes were changed significantly. As expected, the expression of antioxidant enzyme genes were elevated. In the TM6 vs. CK comparison, nine *APX* and six *AOX* genes were also up-regulated. TRINITY_DN4762_c0_g1_i11, which encodes *APX*, was upregulated in all comparisons except in TM48 vs. CK. 17 *CAT* genes were upregulated in the TM24 vs. CK comparison. Among these *CAT* genes, TRINITY_DN139642_c0_g1_i4, TRINITY_DN150689_c0_g1_i1, and TRINITY_DN59405_c0_g1_i1 showed 7.66-, 5.90-, and 5.58-fold upregulated, respectively. In addition, five *DHAR* genes were identified as upregulated DEGs. In the TM12 vs. CK and TM36 vs. CK comparisons, only several selected genes showed significant variations. For the TM48 vs. CK comparison, six *CAT* and one *SOD* genes were significantly upregulated. TRINITY_DN2095_c0_g1_i6, which identified as a NADP reductase, showed 4.46-, 4.84-, 4.81-, 5.12- and 4.37-fold upregulated in TM6 vs. CK, TM12 vs. CK, TM24 vs. CK, TM36 vs. CK, and TM48 vs. CK comparisons.

Some DEGs related to cell wall formation and degeneration including 1,4-β-D-xylan synthase and β-glucosidase were identified. TRINITYs encode glutathione hydrolase, which is a key enzyme involved in cell wall formation, showed upregulated in all comparisons except TM12 vs. CK. TRINITY_DN22409_c0_g1_i6, TRINITY_DN6239_c0_g1_i11, TRINITY_DN14287_c1_g1_i1, and TRINITY_DN4481_c0_g1_i14 in the TM6 vs. CK, TM24 vs. CK, TM36 vs. CK, and TM48 vs. CK showed a 1.91-, 2.50-, 1.95-, and 7.74-fold increase accordingly. TRINITY_DN16008_c0_g1_i4 which annotated as β-glucosidase showed 3.99-, 3.57-, 2.34-, 2.32-, and 3.27-fold increasing in TM6 vs. CK, TM12 vs. CK, TM24 vs. CK, TM36 vs. CK and TM48 vs. CK comparisons, respectively. In addition, TRINITY_DN8969_c0_g1_i8 was annotated as 1,4-β-D-xylan synthase, showing 5.11-, 1.25-, 1.98-, 3.53-, and 4.03-fold increasing in TM6 vs. CK, TM12 vs. CK, TM24 vs. CK, TM36 vs. CK, and TM48 vs. CK comparisons, respectively.

### Validation of the transcriptome analysis by qRT−PCR

**Figure 10:**
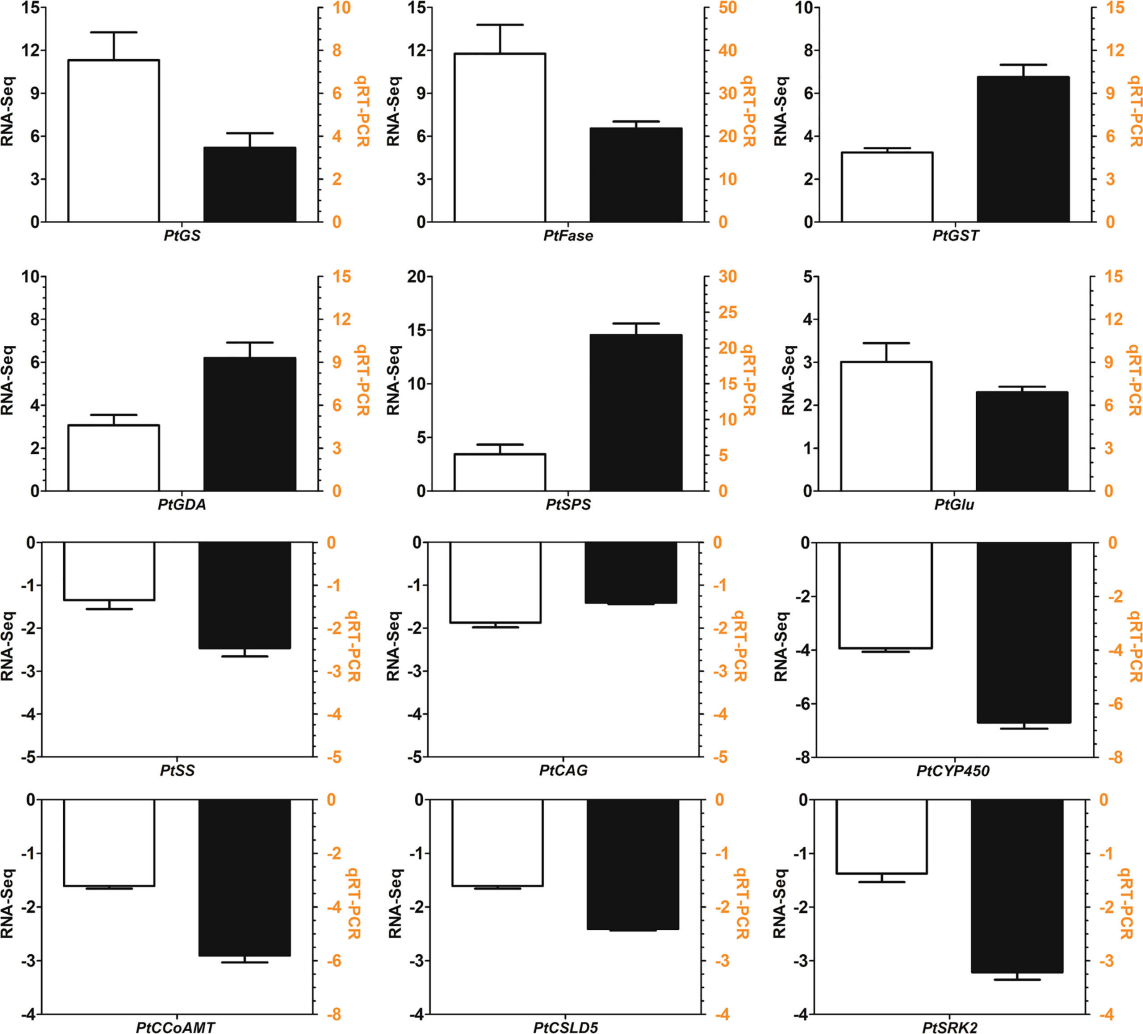
qRT−PCR validation of twelve selected differentially expressed genes (DEGs). The white bar represents the relative expression determined by transcriptome analysis, and the black bar represents the expression level determined by qRT−PCR analysis. *PtCSLD5*: cellulose synthase-like D5; *PtSS*: sucrose synthase; *PtCCoAMT*: caffeic acid 3-O-methyltransferase; *PtCAG*: coniferyl-alcohol glucosyltransferase; *PtSRK2*: serine/threonine-protein kinase SRK2; *PtCYP450*: cytochrome P450 family 98; *PtGlu*: UDP-glucosyl transferase; *PtSPS*: sucrose-phosphate synthase; *PtGST*: glutathione S-transferase; *PtFase*: β-fructofuranosidase; *PtGDA*: glutamate decarboxylase; *PtGS*: glutamate decarboxylase.

To evaluate the validity of the transcriptome analysis, the relative expression profiles of 12 DEGs were verified by qRT*−*PCR. As shown in **Figure 10**, the relative expression of DEGs determined by qRT*−*PCR was consistent with the transcriptome results. qRT*−*PCR analysis showed that the phenylpropanoid biosynthesis genes such as *PtSRK2*, *PtCCoAMT*, *PtCAG* were downregulated. *PtCCoAMT* and *PtCAG* showed 3.31- and 1.22-fold decreases in qRT*−*PCR analysis and the two values were 6.67- and 2.63-fold downregulated in transcriptome analysis. *PtCSLD5* and *PtSS* are two key enzyme genes in starch and sucrose metabolism pathway which encode cellulose synthase-like and sucrose synthase. *PtCSLD5* and *PtSS* showed 1.61- and 1.35-fold downregulation in the transcriptome and these values were 2.41- and 2.64-fold for qRT−PCR (**Figure 10**). A linear regression analysis was also conducted between the transcriptome and qRT−PCR. The regression equation is Y=2.4274X+1.3262 with the R^2^ value is 0.8943. In conclusion, the qRT*−*PCR results not only confirmed the reliability of the transcriptome results but also validated the downregulated genes in the starch and sucrose metabolism and phenylpropanoid biosynthesis pathways.

## Discussion

*P. ternata* is a valuable traditional Chinese medicine and has been used for more than 2000 years in China. *P. ternata* occupies an important position in the traditional Chinese medicine system. It is particularly worth mentioning that *P. ternata* can form Chinese patent medicines with different effects by using different processing methods. For example, the raw *P. ternata* tuber processed with alum is processed into “Qing Banxia”; *P. ternata* processed with fresh ginger and alum are processed into “Jiang Banxia”; and *P. ternata* processed with alum, licorice and lime are processed into “Fa Banxia”. The wild resources of *P. ternata* are on the verge of exhaustion due to the unreasonable artificial collection and degeneration of habitat. In addition, *P. ternata* faces serious CCO and thus has an impeded yield. However, few reports have been conducted to study the allelopathic effects of *P. ternata*. Thus, in this study, physiological and cellular methods combined with transcriptome sequencing were used to clarify the allelopathic effect of phenolic acids on *P. ternata*.

### MPA affected the morphological traits and cell wall integrity

It is well documented that allelochemicals affect the morphological, physiological, and biochemical characteristics of plants. The change in morphological traits is the most direct response to allelochemicals. Thus, we first analyzed the effects of MPA on the morphological traits of *P. ternata* seedlings. The results showed that M-Pa-B (medium concentration) restricted seedling growth, while M-Pa-A (high concentration) resulted in seedling death. *P. ternata* treated with M-Pa-B were withered and wilted which was similar to the phenotype of CCO. Guo et al. (2020) found that cinnamic acid significantly inhibited the growth of faba bean and reduced its biomass.

FDA-PI staining can reflect the survival status of cells in the root tip by staining death cells. FDA could be decomposed by lipases and produces fluorescent polar substances, so viable and intact cells produce green fluorescence. PI cannot pass through living cell membranes, but it can pass through damaged cell membranes and stain nucleus. This method could effectively evaluate the allelopathic effect of allelochemicals. Yang et al. (2018) analyzed the root cell apoptosis of *Panax notoginseng* by FDA-PI staining. After 4 h of ginsenoside treatment, a large number of root cells were held, indicating the allelopathic effect of ginsenoside. Using the PI-staining method, Qiao et al. (2020) indicated that allelochemicals induced root cell death and reflected the effect of allelochemicals on *Panax notoginseng* root cells. Similar to those studies, our results also demonstrated that the cell wall of root cell was damaged or broken after M-Pa treatment.

### MPA changed the physiological and biochemical indices

The variation in the contents of ROS and MDA could offer the results at the physiological level and provide a reference for the in-depth analysis of the response mechanism to allelochemicals. In this study, M-Pa induced a significantly increase in H_2_O_2_, $O_{2}^{-}$ , and MDA contents, suggesting a higher level of ROS and lipid peroxidation. With increasing H_2_O_2_, $O_{2}^{-}$ , and MDA contents, the cell membrane was disrupted, and cell homeostasis was disturbed. Conversely, higher activities of SOD, APX, and DHAR were detected in the M-Pa+As and M-Pa+Ge treatments. The decreased contents of H_2_O_2_, $O_{2}^{-}$ , and MDA were accompanied by increased antioxidant enzyme activity. Based on these results, it can be concluded that M-Pa induced the overaccumulation of H_2_O_2_, $O_{2}^{-}$ , and MDA, and antioxidant enzymes could not scavenge H_2_O_2_ and $O_{2}^{-}$ and lead to cell membrane degeneration and cell death. The protective mechanisms of L-ascorbate acid and β-gentiobiose mediated antioxidant enzyme activity serves to enhance the scavenging ability of ROS, alleviate lipid peroxidation and maintain the cell homeostasis. Through these mechanisms, antioxidants can reduce the toxic effect of phenolic acids on cells. These results were consistent with the previous findings in *Panax notoginseng* (Yang et al., 2018), which reported that the increased ROS can be alleviated by L-ascorbate acid and β-gentiobiose. Guo et al. (2020) demonstrated that cinnamic acid significantly inhibited the defense enzyme activity and reduced the resistance of plants to pathogens, indicating the importance of defense enzymes. In He et al. (2019a) the MDA content in the leaves of *Codonopsis pilosula* planted in a continuous cropping field was significantly higher than that in the control field.

### MPA altered the transcriptional patterns

To investigate the variation in genes expression and pathways enrichment, we analyzed the changes in transcriptome profiles at five time points. To our knowledge, this study provides the largest transcriptome data of *P. ternata* to date. Transcriptome analysis showed that MPA induced the significant variation in transcriptome profiles. GO annotation analysis indicated that functional terms such as “response to oxidative stress”, “response to chemical stimulus” and “metabolic process” were significantly enriched. These results indicated that MPA induced exogenous stress and caused transcriptional variations. Additionally, the expression level of *SOD*, *APX*, and *CAT* were significantly upregulated which supported the higher antioxidant enzyme activity. Several ROS-related DEGs were annotated in “response to reactive oxygen species”, “oxidoreductase activity”, and “cellular response to stress”, which indicating that these DEGs participate in the oxidative damage response to M-Pa treatment. As with our results, Huang et al. (2021) study on sugar beet also indicated that altered DEGs were functionally annotated in pathways related to “oxidoreductase activity”, “oxidation-reduction process”, and “integral component of membrane”. MPA affects the gene expression pattern and therefore induces physiological and phenotypical variations in *P. ternata*.

### Variation in key metabolic pathways in response to MPA

KEGG analysis demonstrated that some DEGs involved in starch and sucrose metabolism and phenylpropanoid biosynthesis were downregulated with M-Pa treatment. Starch and sucrose are essential for plant growth and development, as well as the response to abiotic stress (Ruan, 2014). They are the two main components of *P. ternata* tubers. As the main way for plants to generate and accumulate energy, the sucrose and starch synthesis pathways are usually maintained in a vigorous state. However, Ma-P treatment downregulated key enzyme genes, such as O-glycosyl hydrolase family 17 proteins and glycosyl hydrolase family 32 proteins, resulting in insufficient synthesis of energy substances and reducing resistance to invasion by external diseases and pests. Zeng et al. (2020) study on CCO of *Pogostemon cablin* also indicated that energy metabolism is pivotal for resistance to continuous cropping.

The secondary metabolites of medicinal plants play an important role in stress resistance. Phenylpropanoid biosynthesis is the upstream pathway of the synthesis of flavonoids, phenolic acids, and terpenes. Benzoic acid and ephedrine, two important secondary metabolites in *P. ternata*, are also synthesized through this pathway (Zhang et al., 2016). The DEGs involved in phenylpropanoid biosynthesis, such as *4CL1*, *CCoAMT*, and *C4H* demonstrated lower expression with M-Pa treatment. These results showed that M-Pa treatment reduced the gene expression of key enzymes in the metabolic pathway and affected the accumulation of secondary metabolites, which reduced the synthesis of downstream secondary metabolites and weakened the ability to resist adverse environments and diseases. The results explained the growth inhibition of *P. ternata* with M-Pa treatment at the transcriptome level. Similar to our results, Li et al. (2019) studies *Andrographis paniculata* continuous cropping disorder and demonstrated that the expression levels of several key enzyme genes in the phenylpropanoid biosynthesis pathway decreased significantly. In addition, we noted that plant and pest interactions and plant hormone signal transduction pathways were also changed. TRINITY_DN63533_c0_g1_i4 in the plant hormone signal transduction pathway, which encodes an auxin influx carrier LAX3 that promotes lateral root emergence, showed a 7.81-fold decrease. TRINITY_DN105571_c0_g1_i3, which encodes a cell-wall-remodeling enzyme, showed a 5.39-fold decrease. TRINITY_DN31114_c0_g1_i11, which encodes a transcriptional regulator that represses auxin-inducible gene expression, showed an 8.09-fold decrease. These results suggest that phenolic acids may control the occurrence of fibrous roots and the formation of the cell membrane by regulating the changes in hormone levels. Chi et al. (2013) reported that ferulic acid has a significant effect on inhibiting rice root elongation by modulating ethylene and jasmonic acid. He et al. (2019a) also reported that continuous cropping inhibited the hormone signaling pathways involved in cell division and enlargement of *Codonopsis tangshen*.

## Conclusion

In this study, several biological approaches were integrated to dissect the allelopathic effects of MPA on *P. ternata.* The results demonstrated that MPA has a significant effect on *P. ternata* seedlings and induced remarkable variations at the morphological, physiological, and transcriptome levels. The application of exogenous antioxidants increased the antioxidant enzyme activity, decomposed the excess ROS and decreased the MDA content. In addition, the DEGs encoding key enzyme genes were enriched in starch and sucrose metabolism and phenylpropanoid biosynthesis pathways were significantly downregulated. Taken together, the results of morphological, physiological, and transcriptome analysis suggest that M-Pa leads to excessive ROS accumulation in root cells and causes the cell wall degradation, inducing cell death and root degradation. L-ascorbate acid and β-geniobiose could enhance antioxidant enzyme activity, remove the excess ROS and MDA and thus protect the cell membrane. This study contributes to a further understanding of the physiological and molecular changes of *P. ternata* with mixed phenolic acid treatment. Our results also lay the foundation for resolving the CCO of *P. ternata* and other continuous cropping systems.

## Data availability statement

The data presented in the study are deposited in the NCBI repository,accession number PRJNA791970.

## Author contributions

RM and ZL were the leading investigators of this program. RM and ZH designed the experiments. ZH and RM performed most of experiments and analyzed the data. Other authors assisted in experiments and discussed the results. ZH, YW, and RM wrote the manuscript. All authors approved the submitted version.

## Funding

This work was supported by Natural Science Foundation of China (82260748), the Key R&D Program of the Guangxi Zhuang Autonomous Region (2018AB49021), Natural Science Foundation of Shaanxi Province (2021JQ-636), and Natural Science Foundation of Shaanxi Provincial Department of Education (21JK0989).

## Conflict of interest

The authors declare that the research was conducted in the absence of any commercial or financial relationships that could be construed as a potential conflict of interest.

## Publisher’s note

All claims expressed in this article are solely those of the authors and do not necessarily represent those of their affiliated organizations, or those of the publisher, the editors and the reviewers. Any product that may be evaluated in this article, or claim that may be made by its manufacturer, is not guaranteed or endorsed by the publisher.

## Supplementary material

Supplementary Figure 1 Gene Ontology (GO) classification of the assembled unigenes from the *P. ternata* transcriptome. Unigenes were classified into molecular function (MF), biological process (BP), and cellular component (CC) category

Supplementary Figure 2 Functional classification of KOG analysis.
