# Supplementary figures and images for: Dynamic analysis of physiological indices and transcriptome profiling revealing the mechanisms of the allelopathic effects of phenolic acids on Pinellia ternata
Source: Front Plant Sci. 2022 Oct 18;13:1039507. doi: 10.3389/fpls.2022.1039507 (PMC9635339; doi:10.3389/fpls.2022.1039507)

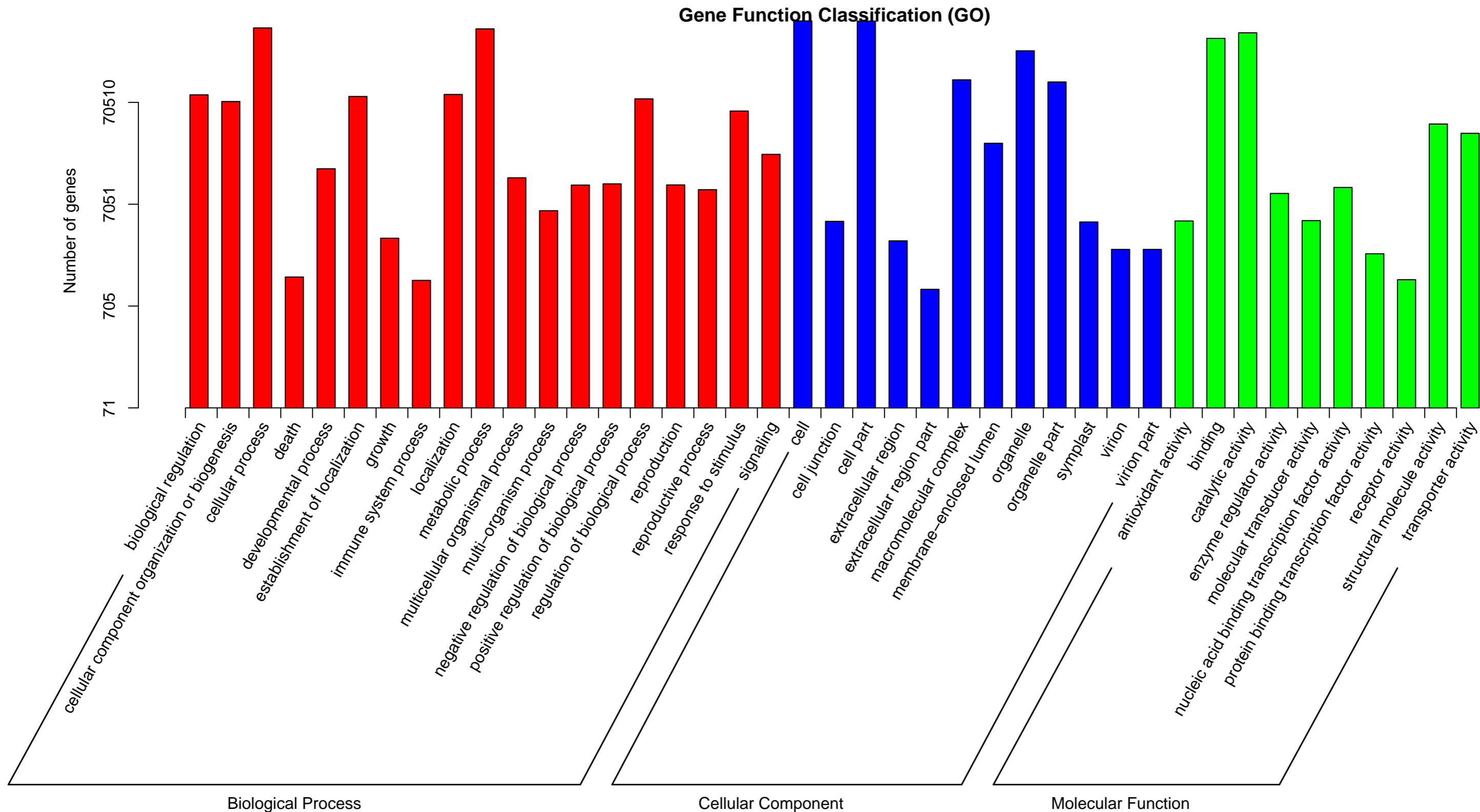

Supplement: Supplementary Figure 1 — Gene Ontology (GO) classification of the assembled unigenes from the P. ternata transcriptome. Unigenes were classified into molecular function (MF), biological process (BP), and cellular component (CC) category [file DataSheet_1.pdf]
