# Supplementary material for: Dynamic analysis of physiological indices and transcriptome profiling revealing the mechanisms of the allelopathic effects of phenolic acids on Pinellia ternata
Source: Front Plant Sci. 2022 Oct 18;13:1039507. doi: 10.3389/fpls.2022.1039507 (PMC9635339; doi:10.3389/fpls.2022.1039507)
Supplement: Supplementary Figure 2 — Functional classification of KOG analysis. [file DataSheet_2.pdf]

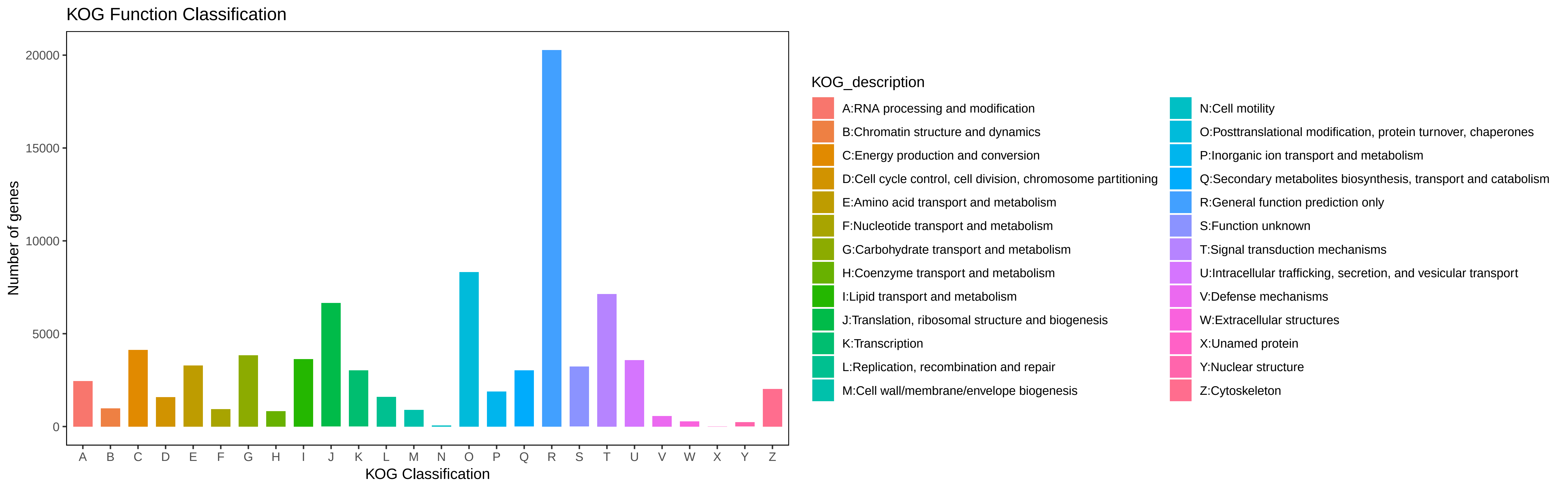
KOG Function Classification
Number of genes
20000
15000
10000
5000
0
A B C D E F G H I J K L M N O P Q R S T U V W X Y Z
KOG Classification
KOG_description
A:RNA processing and modification
B:Chromatin structure and dynamics
C:Energy production and conversion
D:Cell cycle control, cell division, chromosome partitioning
E:Amino acid transport and metabolism
F:Nucleotide transport and metabolism
G:Carbohydrate transport and metabolism
H:Coenzyme transport and metabolism
I:Lipid transport and metabolism
J:Translation, ribosomal structure and biogenesis
K:Transcription
L:Replication, recombination and repair
M:Cell wall/membrane/envelope biogenesis
N:Cell motility
O:Posttranslational modification, protein turnover, chaperones
P:Inorganic ion transport and metabolism
Q:Secondary metabolites biosynthesis, transport and catabolism
R:General function prediction only
S:Function unknown
T:Signal transduction mechanisms
U:Intracellular trafficking, secretion, and vesicular transport
V:Defense mechanisms
W:Extracellular structures
X:Unamed protein
Y:Nuclear structure
Z:Cytoskeleton
