# Supplementary material for: Dynamic analysis of physiological indices and transcriptome profiling revealing the mechanisms of the allelopathic effects of phenolic acids on Pinellia ternata
Source: Front Plant Sci. 2022 Oct 18;13:1039507. doi: 10.3389/fpls.2022.1039507 (PMC9635339; doi:10.3389/fpls.2022.1039507)
Supplement: Supplementary file 3 [file DataSheet_3.docx]

Table S1 The primers used in the present study.

|  | Gene | Forward (5’-3’) | Reverse (5’-3’) |
| --- | --- | --- | --- |
| 1 | *PtSS* | CGGAAGCAAGAGGAACAAAG | GGCGTTGAAGAATCTTGCTC |
| 2 | *PtCcOMA* | GTTCAACGGCAGAGACACAA | AGCGATCTCGGTAGGAGACA |
| 3 | *PtCAG* | CCGGACGAGTCGGTACTTTA | TATGCACTGCAAGCTGAACC |
| 4 | *PtCSLD5* | GGAGAAACGAGCAGTTCTGG | TGGAGGAACCATCAAAAAGC |
| 5 | *PtCAG1* | CCGGACGAGTCGGTACTTTA | TATGCACTGCAAGCTGAACC |
| 6 | *PtCYP450* | AGTGGAGCGAAACAGCATTT | TCGGTTAAGATCCGGTCAAG |
| 7 | *PtGS* | ACGAGACCGCAGACATCAAC | AAGAAGACCCCTCAAGAACCA |
| 8 | *PtGST* | AGGGTGCTGGGAAACTGGAG | CTGGACGATGACGAGGGACT |
| 9 | *PtFase* | GACCGTTTGGCTCAGTAGGG | TCCGCCTGTGATGCGAGAAT |
| 10 | *PtSPS* | GGAGAATGCCGTCCTTTGAG | TTTTGGGTAAGCAACGAGTC |
| 11 | *PtGDA* | ATGTCGGCGTGCCACTCGTA | CTCCTGCTGAAGTCCTCCCT |
| 12 | *PtGlu* | AATACGGAAACCCACCAGTC | CCTCTGACATCACAGCCATC |
| 13 | *PtGAPDH* | TGCTGGGAATGATGTTGAATG | TTGGCATTGTTGAGGGTTTG |

Table S2 Transcriptome profiling of *P. ternata*.

| Sample name | Raw reads | Raw bases | Clean reads | Clean bases | Q30 | Sample name | Raw reads | Raw bases | Clean reads | Clean bases | Q30 |
| --- | --- | --- | --- | --- | --- | --- | --- | --- | --- | --- | --- |
| CK0_1_1 | 28936074 | 4.34G | 27671211 | 4.15G | 93.45% | TM6_1_1 | 29100088 | 4.36G | 27995890 | 4.2G | 93.37% |
| CK0_1_2 | 28936074 | 4.34G | 27671211 | 4.15G | 92.92% | TM6_1_2 | 29100088 | 4.36G | 27995890 | 4.2G | 93.24% |
| CK0_2_1 | 27634704 | 4.14G | 26405983 | 3.96G | 93.37% | TM6_2_1 | 28385076 | 4.25G | 27349810 | 4.1G | 93.68% |
| CK0_2_2 | 27634704 | 4.14G | 26405983 | 3.96G | 92.47% | TM6_2_2 | 28385076 | 4.25G | 27349810 | 4.1G | 93.77% |
| CK0_3_1 | 28196188 | 4.22G | 26961556 | 4.04G | 93.51% | TM6_3_1 | 28048981 | 4.2G | 26991461 | 4.05G | 93.53% |
| CK0_3_2 | 28196188 | 4.22G | 26961556 | 4.04G | 92.51% | TM6_3_2 | 28048981 | 4.2G | 26991461 | 4.05G | 93.46% |
| CK6_1_1 | 25053770 | 3.75G | 24035775 | 3.61G | 94.64% | TM12_1_1 | 26326396 | 3.94G | 25291576 | 3.79G | 93.65% |
| CK6_1_2 | 25053770 | 3.75G | 24035775 | 3.61G | 92.79% | TM12_1_2 | 26326396 | 3.94G | 25291576 | 3.79G | 93.33% |
| CK6_2_1 | 29488295 | 4.42G | 28326368 | 4.25G | 93.52% | TM12_2_1 | 24239765 | 3.63G | 23066935 | 3.46G | 93.10% |
| CK6_2_2 | 29488295 | 4.42G | 28326368 | 4.25G | 92.85% | TM12_2_2 | 24239765 | 3.63G | 23066935 | 3.46G | 92.93% |
| CK6_3_1 | 25738663 | 3.86G | 24604787 | 3.69G | 93.71% | TM12_3_1 | 24424040 | 3.66G | 23104044 | 3.47G | 93.29% |
| CK6_3_2 | 25738663 | 3.86G | 24604787 | 3.69G | 93.87% | TM12_3_2 | 24424040 | 3.66G | 23104044 | 3.47G | 92.14% |
| CK12_1_1 | 27918839 | 4.18G | 26759025 | 4.01G | 93.40% | TM24_1_1 | 31360347 | 4.7G | 30083839 | 4.51G | 93.47% |
| CK12_1_2 | 27918839 | 4.18G | 26759025 | 4.01G | 93.23% | TM24_1_2 | 31360347 | 4.7G | 30083839 | 4.51G | 93.09% |
| CK12_2_1 | 25582922 | 3.83G | 24504886 | 3.68G | 93.45% | TM24_2_1 | 29237427 | 4.38G | 28091395 | 4.21G | 93.52% |
| CK12_2_2 | 25582922 | 3.83G | 24504886 | 3.68G | 91.85% | TM24_2_2 | 29237427 | 4.38G | 28091395 | 4.21G | 92.89% |
| CK12_3_1 | 25134779 | 3.77G | 24076172 | 3.61G | 93.46% | TM24_3_1 | 28801789 | 4.32G | 27602251 | 4.14G | 93.65% |
| CK12_3_2 | 25134779 | 3.77G | 24076172 | 3.61G | 93.39% | TM24_3_2 | 28801789 | 4.32G | 27602251 | 4.14G | 93.43% |
| CK24_1_1 | 29387314 | 4.4G | 28116698 | 4.22G | 93.52% | TM36_1_1 | 30319822 | 4.54G | 28710838 | 4.31G | 93.50% |
| CK24_1_2 | 29387314 | 4.4G | 28116698 | 4.22G | 93.27% | TM36_1_2 | 30319822 | 4.54G | 28710838 | 4.31G | 92.75% |
| CK24_2_1 | 29177424 | 4.37G | 27945196 | 4.19G | 93.60% | TM36_2_1 | 27878613 | 4.18G | 26823857 | 4.02G | 93.39% |
| CK24_2_2 | 29177424 | 4.37G | 27945196 | 4.19G | 93.30% | TM36_2_2 | 27878613 | 4.18G | 26823857 | 4.02G | 93.29% |
| CK24_3_1 | 29290721 | 4.39G | 28203877 | 4.23G | 93.79% | TM36_3_1 | 30572518 | 4.58G | 29421699 | 4.41G | 93.42% |
| CK24_3_2 | 29290721 | 4.39G | 28203877 | 4.23G | 93.01% | TM36_3_2 | 30572518 | 4.58G | 29421699 | 4.41G | 92.68% |
| CK36_1_1 | 25404040 | 3.81G | 24305221 | 3.65G | 93.65% | TM48_1_1 | 27601684 | 4.14G | 26368921 | 3.96G | 93.40% |
| CK36_1_2 | 25404040 | 3.81G | 24305221 | 3.65G | 93.37% | TM48_1_2 | 27601684 | 4.14G | 26368921 | 3.96G | 93.11% |
| CK36_2_1 | 24806299 | 3.72G | 23627542 | 3.54G | 93.48% | TM48_2_1 | 26828143 | 4.02G | 25567466 | 3.84G | 93.59% |
| CK36_2_2 | 24806299 | 3.72G | 23627542 | 3.54G | 93.05% | TM48_2_2 | 26828143 | 4.02G | 25567466 | 3.84G | 93.35% |
| CK36_3_1 | 26563839 | 3.98G | 25383504 | 3.81G | 93.36% | TM48_3_1 | 31467836 | 4.72G | 30009288 | 4.5G | 93.49% |
| CK36_3_2 | 26563839 | 3.98G | 25383504 | 3.81G | 91.97% | TM48_3_2 | 31467836 | 4.72G | 30009288 | 4.5G | 93.25% |
| CK48_1_1 | 27244039 | 4.08G | 26247856 | 3.94G | 93.62% | CK48_2_2 | 27653602 | 4.14G | 26482005 | 3.97G | 93.08% |
| CK48_1_2 | 27244039 | 4.08G | 26247856 | 3.94G | 93.19% | CK48_3_1 | 27126961 | 4.06G | 26050174 | 3.91G | 93.46% |
| CK48_2_1 | 27653602 | 4.14G | 26482005 | 3.97G | 93.40% | CK48_3_2 | 27126961 | 4.06G | 26050174 | 3.91G | 92.54% |

Table S3 Output of transcriptome analysis.

|  | Number of Unigenes | Percentage (%) |
| --- | --- | --- |
| Annotated in NR | 132,819 | 51.53 |
| Annotated in NT | 59,433 | 23.06 |
| Annotated in KO | 48,739 | 18.91 |
| Annotated in SwissProt | 75,613 | 29.34 |
| Annotated in Pfam | 121,017 | 46.95 |
| Annotated in GO | 70,510 | 27.36 |
| Annotated in COG/KOG | 76,556 | 29.70 |
| Annotated in all Databases | 15,209 | 5.90 |
| Annotated in at least one Database | 158,395 | 61.45 |
| Total Unigenes | 257,746 | 100.0 |
